# Sodium–Taste Cells Require *Skn-1a* for Generation and Share Molecular Features with Sweet, Umami, and Bitter Taste Cells

**DOI:** 10.1523/ENEURO.0385-20.2020

**Published:** 2020-12-03

**Authors:** Makoto Ohmoto, Masafumi Jyotaki, J. Kevin Foskett, Ichiro Matsumoto

**Affiliations:** 1 Monell Chemical Senses Center, Philadelphia, PA 19104; 2 Center for Biological Resources and Informatics, Tokyo Institute of Technology, Yokohama 226-8501, Japan; 3 Department of Physiology, University of Pennsylvania, Philadelphia, PA 19104; 4 Department of Cell and Developmental Biology, Perelman School of Medicine, University of Pennsylvania, Philadelphia, PA 19104

**Keywords:** salty, Skn-1a, sodium taste, taste cell

## Abstract

Taste buds are maintained via continuous turnover of taste bud cells derived from local epithelial stem cells. A transcription factor Skn-1a (also known as Pou2f3) is required for the generation of sweet, umami (savory), and bitter taste cells that commonly express TRPM5 and CALHM ion channels. Here, we demonstrate that sodium–taste cells distributed only in the anterior oral epithelia and involved in evoking salty taste also require *Skn-1a* for their generation. We discovered taste cells in fungiform papillae and soft palate that show similar but not identical molecular feature with sweet, umami, and bitter taste-mediated Type II cells. This novel cell population expresses *Plcb2*, *Itpr3*, *Calhm3*, *Skn-1a*, and *ENaC*α (also known as *Scnn1a*) encoding the putative amiloride-sensitive (AS) salty taste receptor but lacks *Trpm5* and *Gnat3*. *Skn-1a*-deficient taste buds are predominantly composed of putative non-sensory Type I cells and sour-sensing Type III cells, whereas wild-type taste buds include Type II (i.e., sweet, umami, and bitter taste) cells and sodium–taste cells. Both *Skn-1a* and *Calhm3*-deficient mice have markedly decreased chorda tympani nerve responses to sodium chloride, and those decreased responses are attributed to the loss of the AS salty taste response. Thus, AS salty taste is mediated by *Skn-1a*-dependent taste cells, whereas amiloride-insensitive salty taste is mediated largely by Type III sour taste cells and partly by bitter taste cells. Our results demonstrate that Skn-1a regulates differentiation toward all types of taste cells except sour taste cells.

## Significance Statement

Salty taste plays an important role in electrolyte homeostasis in body fluids. Other basic tastes are each mediated by specialized sensory cells and elicits either preference or avoidance; in contrast, salty taste elicits both behaviors, depending on concentrations, and is mediated by multiple mechanisms and cell types that are poorly defined. We report that a subset of cells that express ENaCα exhibit a gene expression profile similar but not identical to sweet, umami, and bitter taste cells. They mediate amiloride-sensitive (AS) sodium taste and rely on Skn-1a for their generation and the CALHM ion channel for neurotransmitter release. Amiloride-insensitive (AI) salty taste is partially mediated by sour taste cells, the only taste cells present in the *Skn-1a* knock-out mice.

## Introduction

Individual taste cells mediate one of five basic tastes in mice: sweet, umami (savory), bitter, sour, and salty by sodium salts (Yarmolinsky et al., 2009; Chandrashekar et al., 2010; Matsumoto et al., 2013). Whereas they express their own taste receptors, sweet, umami, and bitter taste cells share an intracellular signal transduction mechanism comprising phospholipase C β2 (PLCβ2), inositol triphosphate receptor type 3 (IP_3_R3), Ca^2+^-dependent monovalent cation channel TRPM5, and voltage-dependent ATP release channel CALHM1/3 (heterooligomeric channel composed of CALHM1 and CALHM3; Liu and Liman, 2003; Zhang et al., 2003; Hisatsune et al., 2007; Taruno et al., 2013; Ma et al., 2018). Sour taste cells have a different molecular signature, specifically expressing *Pkd2l1*, *Otop1*, and Car4 but not *Plcb2* or *Trpm5* (Huang et al., 2006; Chandrashekar et al., 2009; Chaudhari and Roper, 2010; Tu et al., 2018). In contrast, the specific molecular features of the cells that mediate taste evoked by sodium are poorly defined.

Sodium chloride (NaCl) evokes salty taste via amiloride-sensitive (AS) and amiloride-insensitive (AI) mechanisms in taste cells. The AS mechanisms are specific for NaCl and are involved in the attractive responses to NaCl (Chandrashekar et al., 2010; Tordoff et al., 2014; Nomura et al., 2020). In contrast, the AI mechanisms respond to many salts and mediate aversive responses (Oka et al., 2013). The taste cells that mediate AS and AI salt-sensing mechanisms represent distinct populations (Yoshida et al., 2009; Chandrashekar et al., 2010; Roebber et al., 2019). The AS NaCl-sensing taste cells responsible for sodium preference (hereafter referred to as sodium-taste cells) reside in taste buds of fungiform papillae (FuP), but not in circumvallate papillae (CvP), and express the epithelial sodium channel ENaC as the sodium sensor and CALHM1/3 (Chandrashekar et al., 2010; Tordoff et al., 2014; Nomura et al., 2020). In the CvP all taste cells that co-express *Calhm1*, *Calhm3*, and *Trpm5* depend on *Skn-1a* for their generation (Taruno et al., 2013; Ma et al., 2018). Furthermore, *Skn-1a*-deficient mice showed a partial loss of sodium taste responses of gustatory nerves (Larson et al., 2020). Nevertheless, it has been suggested that sodium-taste cells are distinct from Trpm5-expressing (Trpm5^+^) cells and sour taste cells (Chandrashekar et al., 2010). Thus, whereas the specific cell type involved in attractive responses to NaCl share some features with sweet, umami, and bitter taste cells, it appears to be distinct from them. The identity of specific cell types responsible for the aversive AI salt-sensing mechanisms is also uncertain. It has been reported that taste cells that respond to all Cl^–^-containing solutions, including HCl, are AI and lack *ENaCα* expression (Yoshida et al., 2009). Alternately, it has been suggested that AI salt-taste mechanisms reside in bitter taste cells and sour taste cells that express *ENaCα* (Chandrashekar et al., 2010; Oka et al., 2013). In contrast, it was recently proposed that AI NaCl taste resides in sweet and bitter taste cells but not in sour taste cells (Roebber et al., 2019).

Taste cells are epithelial sensory cells that are maintained in taste buds by continuous turnover (Beidler and Smallman, 1965). They are derived from local epithelial stem cells that express *Sox2* and *Krt5* commonly (Stone et al., 1995; Ohmoto et al., 2017, 2020). At the precursor cell stage of taste cell differentiation, a POU homeodomain transcription factor Skn-1a (also known as Pou2f3) specifies the fate of a cell as a sweet, umami, and bitter cell lineage (Matsumoto et al., 2011). Interestingly, Skn-1a is also required for differentiation of putative sensory cells in other tissues including microvillus cells in the main olfactory epithelium, solitary chemosensory cells in the respiratory epithelium, and tuft cells in the intestine, all of which commonly express *Trpm5* similar to sweet, umami, and bitter taste cells (Ohmoto et al., 2013; Yamaguchi et al., 2014; Gerbe et al., 2016). Skn-1a therefore appears to be a master regulator of *Trpm5*-expressing sensory cells (Yamashita et al., 2017).

In the present study, we found that taste cells responsible for AS avidity to NaCl share some molecular features with sweet, umami, and bitter taste cells but are distinct from them. AS responses of the chorda tympani nerve to NaCl are abolished in both *Skn-1a*-deficient and *Calhm3*-deficient mice that also lack perception of sweet, umami, and bitter tastes. The loss of AS neural responses in *Skn-1a*-deficient mice was correlated with the disappearance of taste cells defined by a Calhm3^+^*Trpm5*^–^ molecular identity. Thus, Skn-1a governs the generation of sodium-taste cells in addition to sweet, umami, and bitter taste cells.

## Materials and Methods

### Animals

C57BL/6J (stock #000664) mice were purchased from The Jackson Laboratory. Heterozygous *Skn-1a^+/–^* mice in a 129/B6 mixed background (Matsumoto et al., 2011) were crossed with C57BL/6J mice over 10 generations, and resultant male and female *Skn-1a^+/–^* mice with a C57BL/6J congenic background were crossed to obtain *Skn-1a^–/–^* mice with a C57BL/6J congenic background, which were maintained by crossing homozygous mice. *Calhm3^–/–^* mice have a C57BL/6J background as described previously (Ma et al., 2018). Both sexes were used in all animal experiments, which were conducted according to a protocol approved by the Institutional Animal Care and Use Committee.

### Tissue preparation

For fresh-frozen tissue samples, mice were deeply anesthetized with urethane, and the oral epithelia were dissected and embedded in O.C.T. compound (Sakura Finetech). For tissues fixed with 4% paraformaldehyde (PFA), mice were deeply anesthetized with urethane and transcardially perfused with PBS followed by 4% PFA in PBS. Dissected oral epithelia were postfixed, cryoprotected, and frozen as previously described (Ohmoto et al., 2008). Cryosections (8-μm thickness) were prepared using a Leica CM1900 cryostat (Leica Microsystems), mounted on tissue adhesive-coated glass slides (Fisher Scientific), and preserved at –80°C until use.

### *In situ* hybridization

*In situ* hybridization using fresh-frozen sections was conducted as previously described (Ohmoto et al., 2008; Taruno et al., 2013). Digoxigenin-labeled and fluorescein-labeled antisense RNAs were synthesized and used as probes after fragmentation to ∼150 bases under alkaline conditions. The probe regions are shown in Table 1. Sections were fixed with 4% PFA, treated with diethylpyrocarbonate, prehybridized with salmon testis DNA, and hybridized with the riboprobes for 40 h. After hybridization, the sections were washed in 0.2× SSC. Prehybridization, hybridization, and washing were performed at 58°C except when using the riboprobes for *Calhm1*, *Itpr3*, and *ENaCα*, which were performed at 65°C. After washing, chromogenic and/or fluorescence signals were developed as follows:

**Table 1:** Probes used for *in situ* hybridization analyses

Gene name	Accession no.	Probe region
*ENaCα*	BC133688	913–2333
*Trpm5*	AF228681	310–3491
*Calhm1*	LC270870	1–1407, 2148–2369
*Calhm3*	LC270871	1–1653
*Itpr3*	BC023776	1–3447
*Plcb2*	BC145249	588–3123
*Gnat3*	AK040065	41–1019
*Skn-1a*	NM_011139	72–2363
*Entpd2*	NM_009849	20–1822
*Pkd2l1*	NM_181422	226–3275

For single-label *in situ* hybridization, hybridized probes were detected using alkaline phosphatase-conjugated anti-digoxigenin antibodies (Roche Diagnostics, 11093274910, RRID:AB_514497), and chromogenic signals were developed using 4-nitro blue tetrazolium chloride/5-bromo-4-chloro-3-indolyl phosphate as a substrate for 3 h (to *Plcb2* and *Itpr3*) or two overnights (to *Calhm1*). Stained images were obtained using a Nikon Eclipse 80i microscope (Nikon Instruments) equipped with a DXM1200C digital camera (Nikon).

For double-label fluorescence *in situ* hybridization, the fluorescence signals of the riboprobes were developed using an alkaline phosphatase-conjugated anti-digoxigenin antibody followed by the HNPP Fluorescent Detection set (Roche Diagnostics) and a biotin–conjugated anti-fluorescein antibody (Vector Laboratories, BA-0601, RRID:AB_2336069) followed by an avidin-biotin complex (Vector Laboratories), a TSA Biotin Tyramide Reagent (PerkinElmer), and an Alexa Fluor 488-conjugated streptavidin (Thermo Fisher Scientific). Fluorescence single-plane confocal images were acquired with a Leica TCS SP2 confocal microscope (Leica Microsystems). Optical confocal images were processed with Photoshop (Adobe Systems). For quantification of cells with fluorescence signals, taste buds on every 8, 12, or 16 sections of the FuP and soft palate from three mice were analyzed. For the frequencies of expression of *Skn-1a* and *Entpd2*+*Pkd2l1* in taste bud cells, sections were counterstained with 4',6-diamidino-2-phenylindole (DAPI). The ratios of *Skn-1a*-expressing cells or *Pkd2l1*-expressing or *Entpd2*-expressing cells to the taste bud cells as judged from DAPI and DIC images were calculated using every 8, 12, or 16 sections of the FuP and soft palate of wild-type (WT; *n* = 3) and *Skn-1a^–/–^* (*n* = 3) mice.

For double-labeling of *Calhm1* or *ENaCα* with other genes, fluorescence and chromogenic signals were developed as previously described (Taruno et al., 2013). Prehybridization, hybridization, and washing were performed at 65°C for any probes, and the fluorescence signals were first developed using a biotin-conjugated anti-fluorescein antibody (Vector Laboratories) followed by an avidin-biotin complex (Vector Laboratories), a TSA Biotin Tyramide Reagent (PerkinElmer), and an Alexa Fluor 488-conjugated streptavidin (Thermo Fisher Scientific). After capturing the fluorescence signals with a Leica TCS SP2 confocal microscope (Leica Microsystems), the chromogenic signals of *Calhm1* or *ENaCα* were detected using an alkaline phosphatase-conjugated anti-digoxigenin antibody and 4-nitro blue tetrazolium chloride/5-bromo-4-chloro-3-indolyl phosphate. Stained images were obtained as described above. Fluorescence and stained images were processed with Photoshop (Adobe Systems). For quantification of cells with fluorescence and stained signals, taste buds on every 8, 12, or 16 sections of the FuP and soft palate from three mice were analyzed.

### Immunohistochemistry

Immunohistochemical analyses using 4% PFA-fixed sections were conducted as previously described (Ohmoto et al., 2008; Taruno et al., 2013). The sections were treated in a preheated target retrieval solution (pH 9; Agilent Technologies) at 80°C for 20 min before blocking. Mouse anti-Itpr3 (1:1000, BD Biosciences, 610312, RRID: AB_397704), Rabbit anti-Gnat3 (1:1000, Santa Cruz Biotechnology, sc-395, RRID:AB_673678), anti-T1R3 (1:1000; Ohmoto et al., 2008), anti-Skn-1a (1:1000, Santa Cruz, sc-330, RRID:AB_677443), and anti-dopa decarboxylase (Ddc; 1:2000, GeneTex, GTX30448, RRID:AB_367199) antibodies were used as primary antibodies. Alexa Fluor 488-conjugated goat anti-mouse IgG (1:500, Thermo Fisher Scientific, A-11 029, RRID:AB_2534088) and Alexa Fluor 555-conjugated goat anti-rabbit IgG (1:500, Thermo Fisher Scientific, A-21 429, RRID:AB_2535850) were used as secondary antibodies. Fluorescent images were acquired and processed as described above.

### Whole chorda tympani nerve recordings

We investigated the electrophysiological response of the chorda tympani nerve in mice of the *Skn-1a* knock-out (*Skn-1a*^–/–^) and *Calhm3* knock-out (*Calhm3*^–/–^) strains, using C57BL/6J mice as WT controls (see above). The experimenters were blinded to the genotype of the mice during testing. The mice were anesthetized with an intraperitoneal injection of a mixture of 4.28 mg/ml ketamine, 0.86 mg/ml xylazine, and 0.14 mg/ml acepromazine in saline (5 μl/g body weight). Anesthesia was maintained with additional injections. Each mouse was fixed with a head holder after its trachea was cannulated, and the chorda tympani nerve was dissected free from its junction with the lingual nerve near the tympanic bulla; then the nerve was cut and the central part was placed on a platinum wire recording electrode. An indifferent electrode touched the walls of the wound. Taste stimuli were delivered to the tongue with a computer-controlled open flow system under constant flow and temperature (25°C) conditions. Each stimulation lasted for 30 s with a 60-s rinse between stimulations. Care was taken to ensure that the flow was directed over the FuP. The nerve impulses were fed into a custom-made amplifier, monitored over a loudspeaker and with an oscilloscope, and recorded (PowerLab/sp4; AD Instruments). The integrated response during stimulation was calculated by subtracting the area of nerve activity preceding the stimulation from that during stimulation. Thus, the data reflect the level of activity during the stimulation period. The responses to all compounds were expressed relative to the response to 0.1 m NH_4_Cl, which is derived from solely sour taste cells (Oka et al., 2013), for each mouse as previously described (Matsumoto et al., 2011; Ma et al., 2018). The averages for each animal and group were calculated for the statistical analyses.

### Statistical analyses

Data are shown as the mean ± SEM. A Welch’s *t* test (for histochemical analyses) or repeated-measures two-way ANOVA (for gustatory nerve recordings) was used to determine the effects of genotype using Prism 6 software (GraphPad Software). When a significant interaction was detected between a genotype and a taste solution concentration, Tukey–Kramer multiple comparison tests were conducted to identify significant differences between pairs of mean values.

## Results

Previous studies demonstrated that *Skn-1a* is necessary for the generation of sweet, umami, and bitter taste cells, and that *Calhm1*, *Calhm3*, and *Trpm5* mRNAs are co-expressed only in *Skn-1a*-dependent taste cells in the CvP (Matsumoto et al., 2011; Ma et al., 2018). Intriguingly, *Calhm1* has been implicated in salty taste (Tordoff et al., 2014), whereas it has been suggested that AS NaCl responses arise from cells that lack *Trpm5* expression (Chandrashekar et al., 2010). Recent efforts to identify AS sodium taste cells in the FuP have produced conflicting results. For example, it was suggested that *Calhm3* expressed in *Skn-1a*-dependent taste cells (Ma et al., 2018) is required for AS NaCl responses (Nomura et al., 2020), whereas it has also been suggested that *Skn-1a*-deficient mice that do not express either *Calhm1* or *Calhm3* (Taruno et al., 2013; Ma et al., 2018) still exhibited AS NaCl responses as much as a half of those of WT mice (Larson et al., 2020). To better understand the identity and molecular features of AS NaCl–sensing taste (i.e., as sodium–taste) cells, we conducted *in situ* hybridization analyses in FuP taste buds where sodium–taste cells reside and soft palate and gustatory nerve recordings of the chorda tympani nerve innervating FuP taste buds.

### Taste cell gene expression in taste buds of FuP

**Figure 1.**
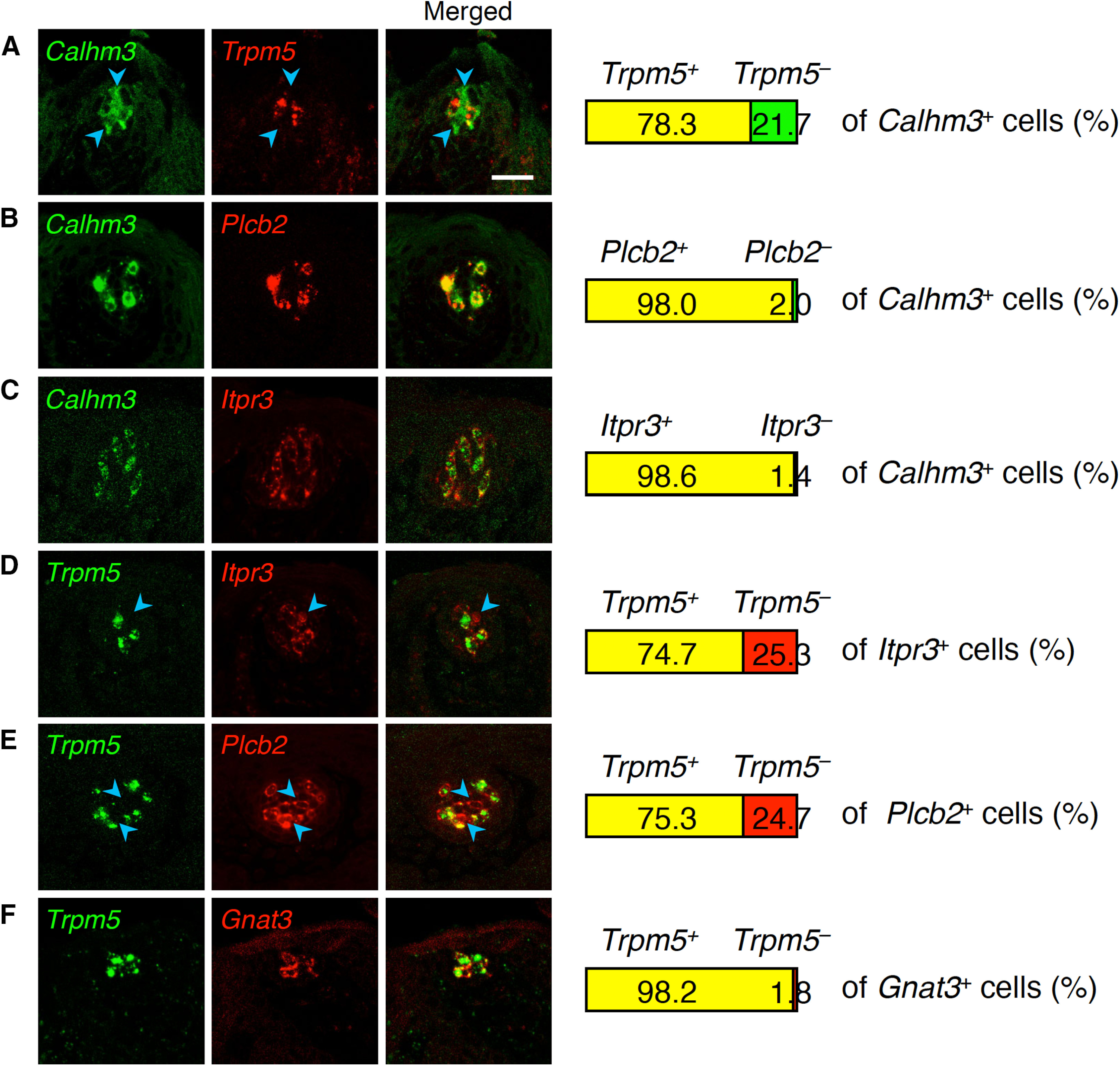
Expression of taste cell genes in taste buds in FuP. Double-fluorescence *in situ* hybridization was performed to study expression of genes required for sweet, umami, bitter, or salty taste perception. Numbers of cells showing signals were counted, and the ratios of cells positive and negative for one gene (***A–C***, middle images; ***D–F***, *Trpm5*) to the total population of cells positive for the other gene (***A–C***, *Calhm3*; ***D–F***, middle image) are shown at the right (*n* = 3). ***A–C***, *Trpm5* (***A***), *Plcb2* (***B***), and *Itpr3* (***C***) compared with *Calhm3*. ***D****–****F***, *Itpr3* (***D***), *Plcb2* (***E***), and *Gnat3* (***F***) compared with *Trpm5*. Blue arrowheads indicate *Calhm3*, *Itpr3*, or *Plcb2* single-positive cells. Scale bar: 25 μm.

First, we asked whether CALHM and Trpm5 channel genes are always co-expressed in the same cells of FuP by double-fluorescence *in situ* hybridization using *Calhm3* and *Trpm5* as probes. In taste buds of FuP where the chorda tympani nerve innervates, *Trpm5* signals were observed only in cells showing *Calhm3* signals. However, ∼20% of *Calhm3*^+^ cells did not generate *Trpm5* signals (Fig. 1*A*). This result is in contrast to their complete co-expression in taste bud cells of the CvP (Ma et al., 2018). Accordingly, these data are consistent with previous findings that sodium taste is mediated by a taste cell subset distinct from sweet, umami, and bitter taste cells but nevertheless requires CALHM channel genes for neurotransmission (Chandrashekar et al., 2010; Tordoff et al., 2014; Nomura et al., 2020).

Then, we examined whether other genes encoding sweet, umami, and bitter taste signaling molecules are expressed in both the *Trpm5*^+^ and *Trpm5*^–^ taste cells. Of note, it has been suggested that sodium–taste cells do not require Ca^2+^ signaling evoked by phosphatidylinositol (PI) turnover, unlike Type II cells (Nomura et al., 2020). Surprisingly, in taste buds of the FuP, *Plcb2* and *Itpr3* were expressed in *Trpm5*^–^ cells and always co-expressed with *Calhm3*, whereas *Gnat3* was expressed only in *Trpm5*^+^ taste cells (Fig. 1). These results strongly suggest that *Calhm3*-expressing taste cells can be classified into two subsets: those that are all positive (i.e., for the expression of *Plcb2*, *Itpr3*, *Calhm3*, *Gnat3*, and *Trpm5*) and those that are *Plcb2*^+^*Itpr3*^+^*Calhm3*^+^*Gnat3*^–^*Trpm5*^–^ (hereafter referred to as *Calhm3*^+^*Trpm5*^–^ cells in this study). In agreement, anti-Itpr3 and anti-Gnat3 antibodies identified Itpr3^+^Gnat3^+^ and Itpr3^+^Gnat3^–^ cells in taste buds of the FuP, whereas in taste buds of the CvP, where cells expressing *Gnat3* and/or *Tas1r3* are identical to Trpm5^+^ cells (Ohmoto et al., 2011), Itpr3^+^ cells are always positive for Gnat3 and/or T1R3 (Extended Data Fig. 1-1*A*). The ratios of Itpr3^+^Gnat3^+^ and Itpr3^+^Gnat3^–^ cells to total Itpr3^+^ cells (Itpr3^+^Gnat3^+^, 77.8%; Itpr3^+^Gnat3^–^ cells, 22.2%) are comparable to those of *Calhm3*^+^*Trpm5*^+^ (78.3%) and *Calhm3*^+^*Trpm5*^–^ (21.7%) cells to *Calhm3*^+^ cells (Fig. 1; Extended Data Fig. 1-1*A*). Consistent with mRNA expression profiles, Itpr3-immunoreactive signals were observed in Skn-1a^+^ cells but not in cells positive for sour taste cell marker Ddc (i.e., DDC^+^ cells; Extended Data Fig. 1-1*B*). Interestingly, cells exhibiting similar molecular features were also detected in taste buds of soft palate that are innervated by the greater superficial petrosal nerves (Extended Data Figs. 1-1, 1-2). Accordingly, the *Calhm3*^+^*Trpm5*^–^ cells found in taste buds in the FuP and soft palate but not in the CvP are predicted to be involved in a taste that is specifically transmitted by the chorda tympani and greater superficial petrosal nerves. Because neurophysiological studies in rats suggest the existence of AS NaCl-sensing taste cells in the greater superficial petrosal nerve-innervated taste buds in soft palate (Harada et al., 1997; Sollars and Hill, 1998), the *Calhm3*^+^*Trpm5*^–^ cells are likely to be sodium–taste cells in the FuP and soft palate taste buds.

10.1523/ENEURO.0385-20.2020.f1-1 Extended Data Figure 1-1 Immunohistochemical identification of sodium–taste cells. **A**, Double-fluorescence immunohistochemistry using anti-Itpr3 and anti-Gnat3 antibodies. Itpr3^+^Gnat3^–^ cells in taste buds of FuP (top) and soft palate (middle) are indicated by blue arrowhead in the merged image (right). In taste buds of CvP (bottom) where cells identified by the expression of Gnat3 and/or T1R3 are equivalent to *Trpm5*^+^ cells (Ohmoto et al., 2011), Itpr3^+^ cells are always positive to Gnat3 and/or T1R3. *N* = 3. **B**, Double-fluorescence immunohistochemistry using anti-Itpr3 and anti-Skn-1a (top) or anti-Ddc (bottom) antibodies. Itpr3 signals are present in Skn-1a^+^ cells and absent in Ddc^+^ cells. *N* = 2. Scale bars: 25 μm. Download Figure 1-1, TIF file.

**Figure 2.**
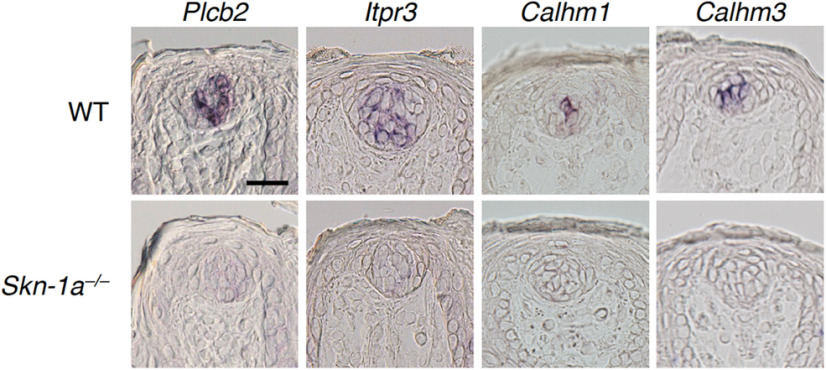
Requirement of *Skn-1a* for the expression of *Plcb2*, *Itpr3*, *Calhm1*, and *Calhm3* in taste buds in FuP. *In situ* hybridization analyses revealed that the expression of *Plcb2*, *Itpr3*, *Calhm1*, and *Calhm3* observed in WT mice (top) were not detected in taste buds in *Skn-1a^–/–^* mice (bottom). Scale bar: 25 μm.

10.1523/ENEURO.0385-20.2020.f1-2 Extended Data Figure 1-2 Expression of taste cell genes in taste buds in soft palate. Double-fluorescence *in situ* hybridization was performed to study the relationship of expression of *Trpm5* with *Calhm3* (***A***), *Itpr3* (***B***), *Plcb2* (***C***), and *Gnat3* (***D***) required for sweet, umami, bitter, or salty taste reception. Numbers of cells showing signals were counted, and the ratios of cells positive and negative for *Trpm5* to the total population of cells positive *Calhm3* (***A***), *Itpr3* (***B***), *Plcb2* (***C***), and *Gnat3* (***D***) are shown at the right (*n* = 3). Blue arrowheads indicate *Calhm3*, *Itpr3*, or *Plcb2* single-positive cells. Scale bar: 25 μm. Download Figure 1-2, TIF file.

**Figure 3.**
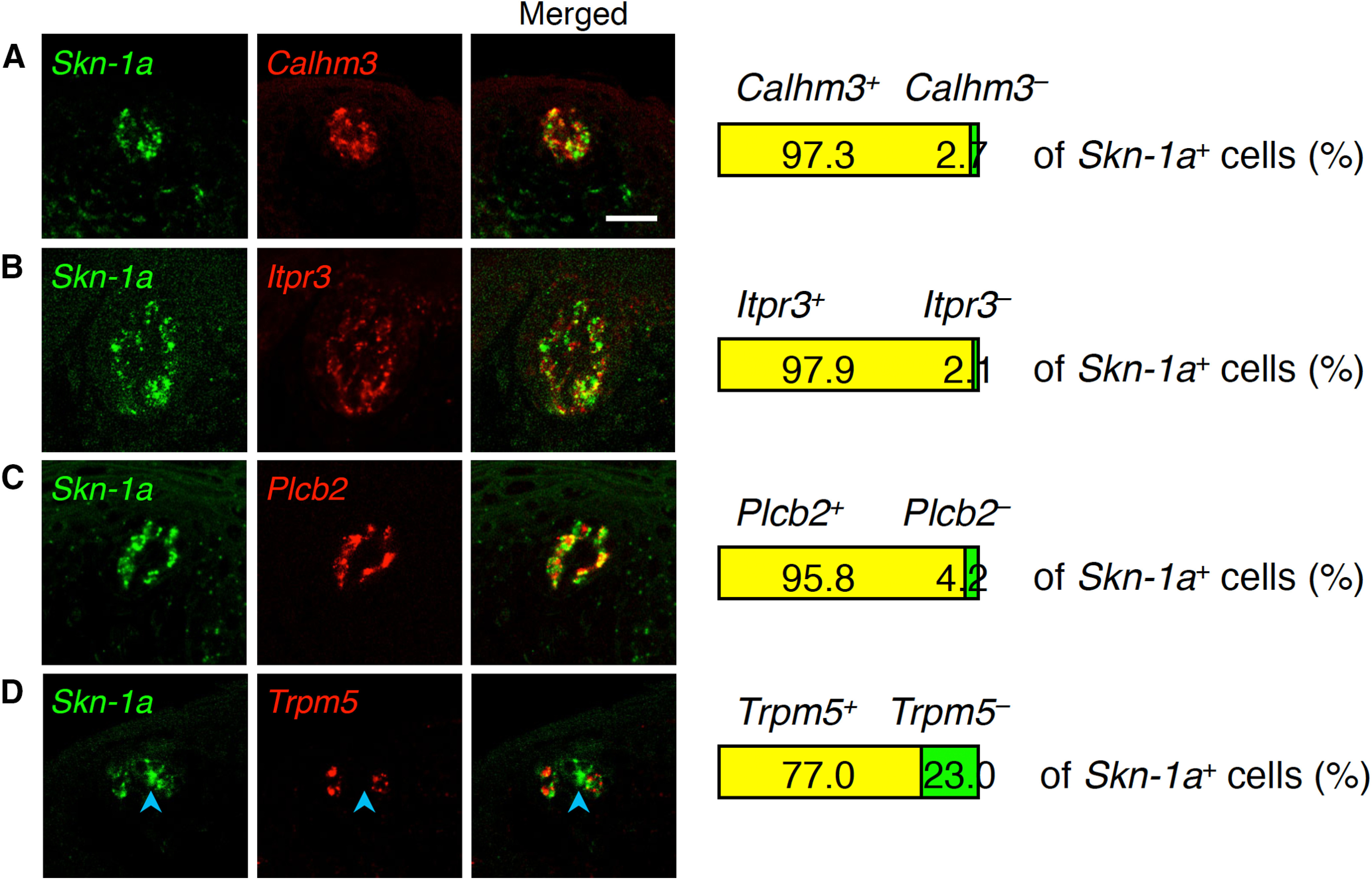
Co-expression of *Skn-1a* with taste cell genes in taste buds in FuP. Double-fluorescence *in situ* hybridization was performed to study the relationship of expression of *Skn-1a* with *Calhm3* (***A***), *Itpr3* (***B***), *Plcb2* (***C***), and *Trpm5* (***D***). Numbers of cells showing signals were counted, and the ratios of cells positive and negative for *Calhm3* (***A***), *Itpr3* (***B***), *Plcb2* (***C***), and *Trpm5* (***D***) to the total population of cells positive for *Skn-1a* are shown at the right (*n* = 3). Blue arrowheads indicate *Skn-1a* single-positive cells. Scale bar: 25 μm.

In taste buds of *Skn-1a*-deficient mice, *Calhm3* expression was not detected in the FuP, palate, or CvP (Ma et al., 2018). Similarly, the expression of *Plcb2*, *Itpr3*, and *Calhm1* was not detected in taste buds in any gustatory areas (Fig. 2; Extended Data Fig. 2-1; Matsumoto et al., 2011; Taruno et al., 2013). Notably, *Plcb2*, *Itpr3*, and *Calhm3* were always co-expressed with *Skn-1a*, and the frequencies of *Skn-1a* signal are comparable to those of *Plcb2*, *Itpr3*, and *Calhm3* signals (Fig. 3; Extended Data Fig. 3-1). Consistent with the relationship of expression of *Trpm5* and *Plcb2*, *Itpr3*, and *Calhm3*, *Trpm5* signals were detected in 77.0 ± 3.2% and 87.2 ± 3.2% of *Skn-1a*^+^ cells in the FuP and soft palate, respectively (Fig. 3; Extended Data Fig. 3-1). These results indicate that *Skn-1a*^+^ cells in the FuP and soft palate can be classified into the same two differentiated cell subsets as *Calhm3*-expressing taste cells: all positive and *Calhm3^+^Trpm5^–^* subsets. The somewhat greater number of cells expressing *Skn-1a* than *Plcb2*, *Itpr3*, and *Calhm3* can be explained by *Skn-1a* expression in putative precursor cells in taste buds in addition to the differentiated taste cells such as sweet, umami, and bitter taste cells.

10.1523/ENEURO.0385-20.2020.f2-1 Extended Data Figure 2-1 Requirement of *Skn-1a* for the expression of *Plcb2*, *Itpr3*, *Calhm1*, and *Calhm3* in taste buds in soft palate. *In situ* hybridization analyses revealed that the expression of *Plcb2*, *Itpr3*, *Calhm1*, and *Calhm3* observed in WT mice (top) were not detected in taste buds in *Skn-1a^–/–^* mice (bottom). Scale bar: 25 μm. Download Figure 2-1, TIF file.

10.1523/ENEURO.0385-20.2020.f3-1 Extended Data Figure 3-1 Co-expression of *Skn-1a* with taste cell genes in taste buds in soft palate. Double-fluorescence *in situ* hybridization was performed to study the relationship of expression of *Skn-1a* with *Calhm3* (***A***), *Itpr3* (***B***), *Plcb2* (***C***), and *Trpm5* (***D***). Numbers of cells showing signals were counted, and the ratios of cells positive and negative for *Trpm5* to the total population of cells positive *Calhm3* (***A***), *Itpr3* (***B***), *Plcb2* (***C***), and *Trpm5* (***D***) are shown at the right (*n* = 3). Blue arrowheads indicate *Skn-1a* single-positive cells. Scale bar: 25 μm. Download Figure 3-1, TIF file.

### Skn-1a is required for generation of *Calhm3*^+^*Trpm5*^–^ cells

**Figure 4.**
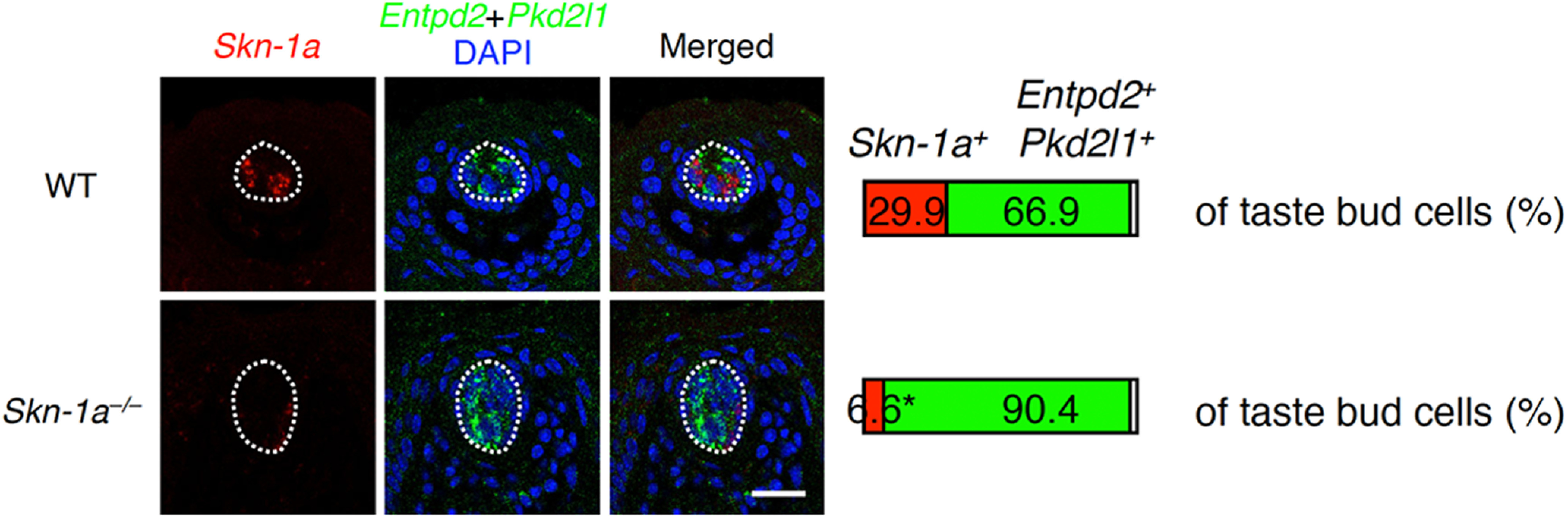
Disappearance of *Skn-1a*-dependent taste bud cells by *Skn-1a* deficiency. Populations of *Skn-1a*^+^ and *Skn-1a*^–^ cells (i.e., positive to a mixed probe to *Entpd2* and *Pkd2l1*) in taste buds in FuP were quantified by double-fluorescence *in situ* hybridization analyses. Taste bud profiles are outlined by broken white line. Asterisk indicates the ratio expressing mutant *Skn-1a* mRNA. The decrease of the *Skn-1a*^+^ cell population was statistically evaluated by Welch’s *t* test: *p *=* *0.0056. Scale bar: 25 μm.

*Skn-1a* is required for the generation of Type II cells (based on morphologic classification) that express *Trpm5* and *Plcb2* and mediate sweet, umami, and bitter tastes (Matsumoto et al., 2011). The *Calhm3^+^Trpm5^–^* cells also express *Skn-1a* (Figs. 2, 3; Extended Data Figs. 2-1, 3-1). Thus, we asked whether *Skn-1a* is required for the generation of *Calhm3*^+^*Trpm5*^–^ cells or simply for the expression of *Plcb2*, *Itpr3*, and *Calhm3* in the *Calhm3*^+^*Trpm5*^–^ cells independent of their generation. For this, we employed double-label fluorescence *in situ* hybridization analyses using probes to detect *Skn-1a*^+^ and *Skn-1a*^–^ taste bud cells. Because *Skn-1a*^–^ taste bud cells are comprised predominantly of Type I putative non-sensory cells and Type III sour taste cells, we used probes for *Entpd2* that is expressed in Type I cells and for *Pkd2l1* that is expressed in sour taste cells to identify *Skn-1a*^–^ cells. In WT mice, FuP taste buds contained 66.9 ± 2.4% *Skn-1a*^–^ (i.e., *Entpd2*-*Pkd2l1* mixed probe–positive) cells and 29.9 ± 3.0% *Skn-1a*^+^ cells (Fig. 4). In the *Skn-1a*-deficient mice, the taste buds contained 90.4 ± 1.1% *Skn-1a*^–^ (i.e., *Entpd2* and *Pkd2l1* mixed probe-positive) cells, and 6.6 ± 1.8% *Skn-1a*^+^ cells (*p *=* *0.0056; Fig. 4). *Skn-1a*-deficient mice express mutant *Skn-1a* mRNA in basal cells, putatively postmitotic taste bud precursor cells (Matsumoto et al., 2011), that likely accounts for the residual *Skn-1a* mRNA signal. These results demonstrate that *Skn-1a*-deficient mice lack all *Skn-1a*^+^ differentiated cells including *Calhm3*^+^*Trpm5*^–^ cells. Similar results were observed in taste buds of soft palate (32.4 ± 0.7% and 5.5 ± 1.1% *Skn-1a*^+^ cells in WT and *Skn-1a*-deficient mice, respectively, *p *=* *0.0001; Extended Data Fig. 4-1). Thus, like sweet, umami, and bitter Type II taste cells, *Calhm3*^+^*Trpm5*^–^ cells also require *Skn-1a* for their generation.

10.1523/ENEURO.0385-20.2020.f4-1 Extended Data Figure 4-1 Disappearance of *Skn-1a*-dependent taste bud cells by *Skn-1a* deficiency. Populations of *Skn-1a*^+^ and *Skn-1a*^–^ cells (i.e., positive to a mixed probe to *Entpd2* and *Pkd2l1*) in taste buds in soft palate were quantified by double-fluorescence *in situ* hybridization analyses. Taste bud profiles are outlined by broken white lines. Asterisk indicates the ratio expressing mutant *Skn-1a* mRNA. Decrease of *Skn-1a*^+^ cell population was statistically evaluated by Welch’s *t* test: *p *=* *0.0001. Scale bar: 25 μm. Download Figure 4-1, TIF file.

### Skn-1a is required for gustatory nerve responses to NaCl

**Figure 5.**
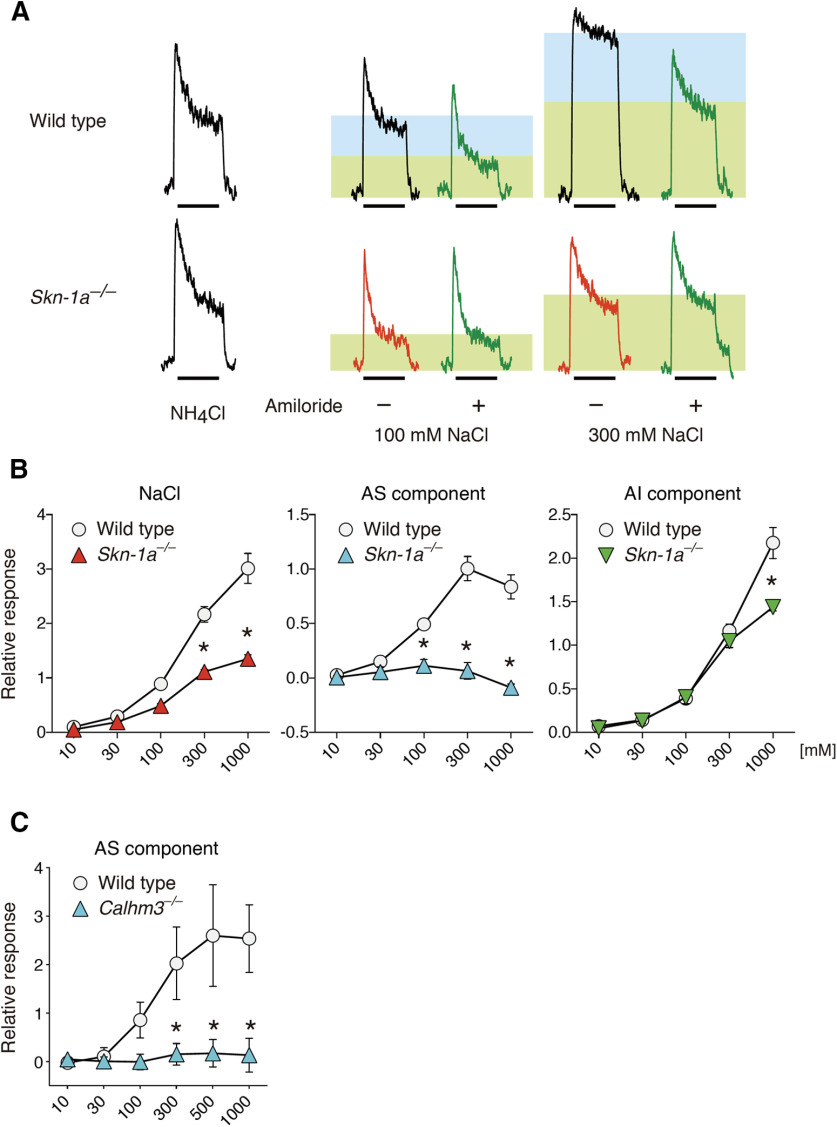
*Skn-1a* deficiency extinguishes AS chorda tympani nerve responses to NaCl. ***A***, Representative charts of chorda tympani nerve responses of WT and *Skn-1a^–/–^* mice to NaCl in the presence (green traces) or absence of 100 μm amiloride. Shaded rectangles depict the AS (blue) and AI (green) components in response to NaCl. The bars under the traces show the duration (30 s) of the taste stimulus. ***B***, ***C***, Whole chorda tympani nerve responses of *Skn-1a^–/–^* (*n* = 3) and WT (*n* = 4) mice (***B***) and *Calhm3^–/–^* (*n* = 6) and WT (*n* = 5) mice (***C***) to NaCl. AS salt responses (AS component; ***B***, middle) were measured by subtracting the AI response (AI component; ***B***, right) from the whole salt response (***B***, left). Significance was assessed by a repeated-measures two-way ANOVA and the Tukey–Kramer test: *p <* *0.05. Data are expressed as the mean ± SEM; where error bars are not visible, they are smaller than the symbol depicting the mean. For details, see Table 2.

Because the existence of *Calhm3*^+^ cells depends on *Skn-1a*, the phenotype by *Calhm3* deficiency in gustatory nerve responses is expected to be recapitulated in the *Skn-1a*-deficient mice. However, recent findings by two groups presented conflicting results, although they both showed the requirement, at least in part, of *Calhm3* and *Skn-1a* in the AS NaCl responses of chorda tympani nerves (Larson et al., 2020; Nomura et al., 2020). We interrogated whether *Skn-1a* and *Calhm3* are equally required for NaCl taste by recording chorda tympani nerve responses. Genetic deletion of *Skn-1a* reduced responses to 300 and 1000 mM NaCl (*p *<* *0.0001). AS responses were eliminated at all concentrations over 100 mM (*p *<* *0.0001 for 100, 300, and 1000 mM), whereas AI responses were diminished only at 1000 mM (*p *<* *0.0001; Fig. 5*A*,*B*; Table 2). The decrease of AI responses in the *Skn-1a*-deficient mice can most likely be accounted for by the absence of bitter taste cells in these mice that contribute to the taste of high salt concentrations (Matsumoto et al., 2011; Oka et al., 2013). These results demonstrate that both AS and AI NaCl tastes are largely mediated by *Skn-1a*-dependent taste bud cells. Consistent with this, AS responses of the chorda tympani nerve to NaCl were eliminated in *Calhm3*-deficient mice (Fig. 5*C*), and *Calhm3* is involved in AI chorda tympani nerve responses in bitter taste cells (Ma et al., 2018). Together, our results indicate that the *Calhm3*^+^*Trpm5*^–^ cells mediate sodium taste, whereas sour taste cells together with bitter taste cells mediate AI salt taste, as previously demonstrated (Oka et al., 2013). These results support the finding by Nomura et al. (2020) and are partially consistent with the results by Larson et al. (2020), with regard to AS NaCl responses. Furthermore, they question the claim that AI NaCl taste is mediated by Type II cells including sweet taste cells (Roebber et al., 2019).

**Table 2:** Summary of statistical analyses of chorda tympani responses to NaCl

Component	Genotype		Concentration		Interaction		Concentrations differing significantly with *p* value
*Skn-1a* KO vs WT							
Whole to NaCl	*F*_(1,5)_ = 42.156	*p* = 0.0013	*F*_(4,20)_ = 138.85	*p* < 0.0001	*F*_(4,20)_ = 19.995	*p* < 0.0001	300 mM (*p* < 0.0001)
							1000 mM (*p* < 0.0001)
AS	*F*_(1,5)_ = 44.941	*p* = 0.0011	*F*_(4,20)_ = 34.275	*p* < 0.0001	*F*_(4,20)_ = 38.247	*p* < 0.0001	100 mM (*p* = 0.0149)
							300 mM (*p* < 0.0001)
							1000 mM (*p* < 0.0001)
AI	*F*_(1,5)_ = 10.019	*p* = 0.0249	*F*_(4,20)_ = 174.55	*p* < 0.0001	*F*_(4,20)_ = 8.0069	*p* = 0.0005	1000 mM (*p* < 0.0001)
*Calhm3* KO vs WT							
AS	*F*_(1,9)_ = 35.852	*p* = 0.0002	*F*_(5,45)_ = 47.097	*p* < 0.0001	*F*_(5,45)_ = 37.400	*p* < 0.0001	300 mM (*p* < 0.0001)
							500 mM (*p* < 0.0001)
							1000 mM (*p* < 0.0001)

### *Calhm3*^+^*Trpm5*^–^ cells express *ENaCα*

Sodium taste deficiency by the conditional deletion of *ENaCα* in *Calhm3*^+^ taste cells (Nomura et al., 2020) and the lack of Trpm5 expression in the putative *ENaCα*^+^ cells identified by a reporter expression in transgenic mice (Chandrashekar et al., 2010) suggest that *ENaCα* is expressed in *Calhm3*^+^*Trpm5*^–^ cells. However, it was not confirmed that the reporter recapitulates the *ENaCα* expression in the FuP and/or soft palate, since reginal expression may be regulated by a distinct enhancer that may not be included in the transgene, as shown for *Shh* expression (Sagai et al., 2009). Thus, we tested the possibility that *ENaCα* is expressed in *Calhm3*^+^*Trpm5*^–^ cells.

**Figure 6.**
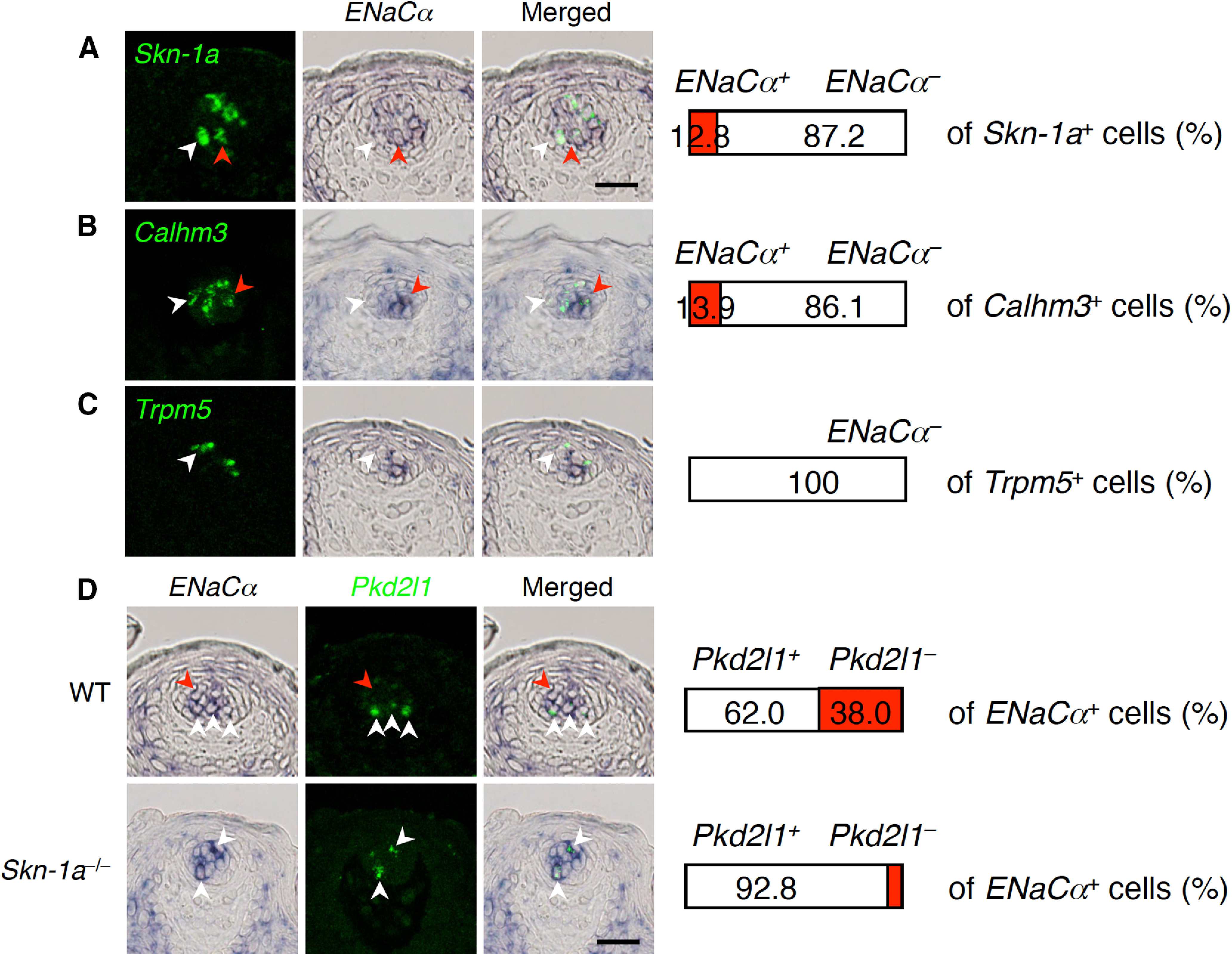
*ENaCα* expression in *Calhm3*^+^*Trpm5*^–^ sodium–taste cells in FuP. Double-labeling *in situ* hybridization was performed to study expression of *ENaCα* in *Calhm3*^+^*Trpm5*^–^ sodium–taste cells. ***A****–****C***, *ENaCα* expression and that of *Skn-1a* (***A***), *Calhm3* (***B***), and *Trpm5* (***C***). Numbers of cells showing signals were counted, and the ratios of cells positive and negative for *ENaCα* (middle images) to the total population of cells positive for the gene (left images) are shown at the right (*n* = 3). White arrowheads indicate *Skn-1a*, *Calhm3*, or *Trpm5* single-positive cells, and red arrowheads indicate cells co-expressing *ENaCα* and *Skn-1a*, *Calhm3*, or *Trpm5*. ***D***, Robust decrease of *ENaCα*-expression in non-sour taste cells by *Skn-1a* deficiency in taste buds. Populations of *Pkd2l1*^+^ and *Pkd2l1*^–^ cells in *ENaCα*-expressing cells were quantified by double-labeling *in situ* hybridization analyses. White and red arrowheads indicate representative *Pkd2l1*^+^*ENaCα*^+^ and *Pkd2l1*^–^*ENaCα*^+^ cells, respectively. Decrease of the *Pkd2l1*^–^*ENaCα*^+^ cell population was evaluated by Welch’s *t* test: *p *=* *0.0422. Scale bars: 25 μm.

Because *ENaCα* signals in taste buds in the FuP were too weak to detect by double-fluorescence *in situ* hybridization, we employed long-term signal development using chromogenic substrate to detect *ENaCα* expression in combination with fluorescence signal detection for other taste cell genes. This method was previously shown to be as efficacious as double-fluorescence *in situ* hybridization in an analysis of the relationship of weak *Calhm1* expression with taste cell marker gene expression (Taruno et al., 2013). Employing this method, we found that *Calhm1* was always co-expressed with *Calhm3* (Extended Data Fig. 6-1), consistent with the same phenotypes of the knock-outs in gustatory nerve recordings (Fig. 5; Taruno et al., 2013; Tordoff et al., 2014; Ma et al., 2018; Nomura et al., 2020). Similarly, we observed partial overlap of *ENaCα* with *Skn-1a* and *Calhm3* localizations (Fig. 6*A*,*B*; Extended Data Fig. 6-2*A*,*B*; Table 3). Although fluorescence signals of a cell may possibly overlap with chromogenic signals of another cell located above or below of fluorescence signal^+^ cell, we never observed any overlap of *ENaCα* with *Trpm5* in the FuP or soft palate (Fig. 6*C*; Extended Data Fig. 6-2*C*; Table 3), strongly suggesting that *ENaCα* and *Trpm5* are not co-expressed in any taste cells. In addition, *ENaCα* expression was detected in both sour and non-sour taste cells (Fig. 6*D*), consistent with previous findings in transgenic mice (Chandrashekar et al., 2010) but incompatible with a previous single cell-PCR analysis (Yoshida et al., 2009). The *ENaCα* expression in non-sour taste cells was absent in *Skn-1a*-deficient mice (in FuP, *p *=* *0.0422; in soft palate, *p *=* *0.0009; Fig. 6; Extended Data Fig. 6-2; Table 3). These results indicate that Skn-1a-dependent *Calhm3*^+^*Trpm5*^–^ cells express *ENaCα* and serve as sodium–taste cells.

**Table 3:** Details of double-label *in situ* hybridization analyses for *ENaC*α with taste cell marker genes

Tissue	Mouse	Marker gene	No. of taste buds	Ratio (%)*
FuP	B6, *n* = 3	*Skn-1a*	40	12.8 ± 2.2
	B6, *n* = 3	*Calhm3*	25	13.9 ± 3.2
	B6, *n* = 3	*Trpm5*	27	0
	B6, *n* = 3	*Pkd2l1*	20	38.0 ± 7.2
	*Skn-1a*^–/–^, *n* = 3	*Pkd2l1*	27	7.2 ± 2.0
Palate	B6, *n* = 3	*Skn-1a*	32	13.0 ± 4.7
	B6, *n* = 3	*Calhm3*	28	8.8 ± 0.6
	B6, *n* = 3	*Trpm5*	25	0
	B6, *n* = 3	*Pkd2l1*	22	44.5 ± 3.0
	*Skn-1a*^–/–^, *n* = 3	*Pkd2l1*	39	2.9 ± 1.7

Double-positive cells to marker gene-positive cells (*Skn-1a*, *Calhm3*, and *Trpm5*) or to *Pkd2l1*^–^*ENaCα*^+^ cells to *ENaCα^+^* cells.

10.1523/ENEURO.0385-20.2020.f6-1 Extended Data Figure 6-1 Co-expression of *Calhm1* and *Calhm3* in taste buds. Double-fluorescence *in situ* hybridization was performed to study the relationship of expression of *Calhm1* with *Calhm3*. Numbers of cells showing signals were counted, and the ratios of cells positive and negative for *Calhm1* to the total population of cells positive *Calhm3* are shown at the right (*n* = 3). Scale bars: 25 μm. Download Figure 6-1, TIF file.

10.1523/ENEURO.0385-20.2020.f6-2 Extended Data Figure 6-2 *ENaCα* expression in *Calhm3*^+^*Trpm5*^–^ sodium–taste cells in soft palate. Double-labeling *in situ* hybridization was performed to study expression of *ENaCα* in *Calhm3*^+^*Trpm5*^–^ sodium–taste cells. ***A****–****C***, *ENaCα* expression and that of *Skn-1a* (***A***), *Calhm3* (***B***), and *Trpm5* (***C***). Numbers of cells showing signals were counted, and the ratios of cells positive and negative for *ENaCα* (middle images) to the total population of cells positive for the gene (left images) are shown at the right (*n* = 3). White arrowheads indicate *Skn-1a*, *Calhm3*, or *Trpm5* single-positive cells, and red arrowheads indicate cells co-expressing *ENaCα* with *Skn-1a*, *Calhm3*, or *Trpm5*. ***D***, Robust decrease of *ENaCα*-expression in non-sour taste cells by *Skn-1a* deficiency in taste buds. Populations of *Pkd2l1*^+^ and *Pkd2l1*^–^ cells in *ENaCα*-expressing cells were quantified by double-labeling *in situ* hybridization analyses. White and red arrowheads indicate representative *Pkd2l1*^+^*ENaCα*^+^ and *Pkd2l1*^–^*ENaCα*^+^ cells, respectively. Decrease of the *Pkd2l1*^–^*ENaCα*^+^ cell population was evaluated by Welch’s *t* test: *p *=* *0.0009. Scale bars: 25 μm. Download Figure 6-2, TIF file.

## Discussion

The results of the present study demonstrate that sodium–taste cells and Type II sweet, umami, and bitter taste cells have shared molecular expression features, and a similar reliance on *Skn-1a* for their generation. These findings advance our understanding of the molecular mechanisms of taste cell differentiation, provide new insights into classification of taste cell lineage, and reveal a cellular mechanism that elicits salt taste.

### Cellular mechanism of taste by NaCl

Salts are dissolved in saliva, and either cations or anions could activate different taste cells independently. In case of NaCl, Na^+^ activates ENaCα–mediated AS mechanisms in a specific population of taste cells characterized by their *ENaCα*^+^*Pkd2l1*^–^*Trpm5*^–^ expression profile (Chandrashekar et al., 2010). Although it has been suggested that these AS salt taste cells do not possess voltage–gated Na^+^ currents (Vandenbeuch et al., 2008), several studies have demonstrated that sodium–taste cells fire Na^+^ action potentials (Bigiani and Cuoghi, 2007; Yoshida et al., 2009; Nomura et al., 2020) and that they are responsible for the avidity to NaCl (Chandrashekar et al., 2010; Nomura et al., 2020). They are most likely sensory cells.

Various chloride salts are sensed by yet-to-be identified AI mechanisms that may reside in sour and bitter taste cells (Oka et al., 2013) or sweet and bitter taste cells (Roebber et al., 2019). If AI salt taste resides in only sweet and bitter taste cells, chorda tympani nerve responses to NaCl in mice deficient in *Calhm1*, *Calhm3*, and *Skn-1a* should be absent over all concentrations. However, chorda tympani nerve responses to NaCl in these mice are comparable to those of WT mice except at very high concentrations (Fig. 5; Tordoff et al., 2014; Ma et al., 2018). Of note, *Skn-1a*-deficient mice have only sour taste cells as sensory cells, and retain AI salt taste, demonstrating that the AI mechanism resides at least in part in sour taste cells. Consistent with this, Car4, which is expressed specifically in sour taste cells in taste buds, is involved in sensing a variety of chloride salts, although the mechanisms are unclear (Oka et al., 2013). Bitter taste cells have also been implicated in aversive salt taste (Oka et al., 2013). Mice deficient in any intracellular bitter signal transduction pathway molecule including Gnat3, PLCβ2, Trpm5, CALHM1, and CALHM3 exhibit deficits in neural or behavioral responses to high salt concentrations (Dotson et al., 2005; Glendinning et al., 2005; Oka et al., 2013; Taruno et al., 2013; Ma et al., 2018). It is possible that T2R bitter receptors associated with Gnat3 respond to chloride salts and trigger the bitter receptor downstream intracellular signal transduction pathway. Of note, human TAS2R7 serves as metallic cation receptor (Behrens et al., 2019; Wang et al., 2019). It is interesting to speculate that specific receptors for Cl^–^ exist in sour and bitter taste cells that respond to various chloride salts.

### Intracellular signal transduction of sodium taste

Our results suggest that taste bud cells in the FuP with the *ENaCα*^+^*Pkd2l1*^–^*Trpm5*^–^ expression profile function as taste receptor cells responsible for sensing Na^+^ (Chandrashekar et al., 2010). Similar to other tastes, NaCl taste involves ATP release in neurotransmission (Finger et al., 2005), and deficiencies of *Calhm1* and *Calhm3* encoding functional components of the ATP-release channel eliminate AS salt responses (Fig. 5; Tordoff et al., 2014; Nomura et al., 2020). Sodium–taste cells also express *Plcb2* and *Itpr3* (Fig. 1), both of which are involved in the increases of intracellular [Ca^2+^] in sweet, umami, and bitter Type II taste cells (Zhang et al., 2003). However, unlike their involvement in Type II cells, neither PLCβ2 nor IP_3_R3 is involved in the perception of NaCl (Zhang et al., 2003; Hisatsune et al., 2007; Tordoff and Ellis, 2013), consistent with recent findings that sodium–taste cells fire action potentials without increases of intracellular [Ca^2+^] (Nomura et al., 2020). The roles of PLCβ2 and IP_3_R3 in sodium–taste cells remain to be determined. Although sodium–taste cells lack expression of *Gnat3* and *Trpm5*, their expression of *Plcb2* and *Itpr3* in sodium–taste cells may suggest that they express yet-to-be-identified G protein-coupled receptor (GPCR), G proteins, and cation channels (possibly Ca^2+^-dependent monovalent cation channels like Trpm5). It is therefore of some interest to understand the transcriptome of sodium–taste cells.

### Sodium–taste cells and the morphologic classification of taste bud cells

Skn-1a regulates the differentiation of sweet, umami, and bitter taste cells and extra-oral taste cell-like chemosensory cells such as brush cells in the airways, urethra, and auditory tube. Like taste cells, those chemosensory cells express taste GPCRs (i.e., T1Rs and/or T2Rs), Gnat3, PLCβ2, and Trpm5 (Finger et al., 2003; Ohmoto et al., 2008; Krasteva et al., 2011, 2012; Matsumoto et al., 2011; Deckmann et al., 2014; Panneck et al., 2014; Yamashita et al., 2017). On the other hand, microvillous cells in the main olfactory epithelium have little similarity to taste and taste cell-like chemosensory cells with regard to molecular feature: they express only Trpm5 but not mRNA of taste GPCRs, Gnat3, or PLCβ2 (Yamaguchi et al., 2014), although immunoreactivities to Gnat3 and PLCβ2 were somehow detected (Genovese and Tizzano, 2018). Although neither intestinal tuft cells nor olfactory microvillous cells express taste GPCRs, the former express another GPCR, Sucnr1, and are involved in sensing chemical succinate, and the latter likely detect odor chemicals and modulate olfactory sensory neuron activity (Lemons et al., 2017; Lei et al., 2018; Nadjsombati et al., 2018; Schneider et al., 2018). The only commonality among these Skn-1a-dependent chemosensory cells, including taste cells, is their expression of Trpm5 (Yamashita et al., 2017). Therefore, sodium–taste cells are the first, very unique population of Skn-1a-dependent chemosensory cells that lack of *Trpm5* expression. Other unidentified Skn-1a-dependent chemosensory cells devoid of *Trpm5* expression may exist. Genetic tools to mark Skn-1a^+^ cells will help identify such novel chemosensory cells.

Taste bud cells have been classified into four types based on their ultra-microscopic morphologic features. This morphologic classification correlates with molecular features: Type I cells appear to be non-sensory supporting cells that lack voltage-gated Na^+^ currents (Medler et al., 2003) and express Entpd2 that hydrolyzes extracellular ATP released from other taste bud cells as a neurotransmitter (Finger et al., 2005; Bartel et al., 2006; Vandenbeuch et al., 2013); Type II cells are taste cells expressing GPCRs and signaling molecules including PLCβ2, IP_3_R3, and Trpm5; Type III cells are Pkd2l1^+^ cells; and Type IV cells are undifferentiated basal cells (Yang et al., 2000; Clapp et al., 2004; Chaudhari and Roper, 2010). Sodium–taste cells are sensory cells, but they are distinct from these cell types. Their molecular features, including the expression of *Plcb2*, *Itpr3*, *Calhm1*, *Calhm3*, and *Skn-1a*, requirement of *Skn-1a* for their generation, and apparent *Calhm1/3* requirement for neurotransmitter release are reminiscent of Type II cells. They are however distinguished from Type II cells by their lack of *Trpm5* and presence of *ENaCα* expression. Accordingly, sodium–taste cells can be regarded as a Type II cell subset, similar to the distinctions among Type II cells by their GPCR expression profiles. Ultramicroscopic morphologic studies in combination with immunohistochemistry in FuP taste buds where sodium–taste cells reside will be necessary to determine whether sodium–taste cells constitute a morphologically-distinct cell type in taste buds.

## Synthesis

Reviewing Editor: Christina Zelano, Northwestern University Decisions are customarily a result of the Reviewing Editor and the peer reviewers coming together and discussing their recommendations until a consensus is reached. When revisions are invited, a fact-based synthesis statement explaining their decision and outlining what is needed to prepare a revision will be listed below. The following reviewer(s) agreed to reveal their identity: Hojoon Lee.

Both reviewers agreed that this study is very interesting, and has the potential to contribute a significant step forward in the field. However, reviewers also pointed out some weaknesses, and agreed on 3 major necessary revisions:

1. First, the reviewers pointed out that this same Skn1a KO mouse line has been tested for salty taste by 3 previous studies (Matsumoto, 2011; Larson, 2020; this study), and different conclusions have been reached every time. However, this study tested the amiloride-sensitive component of salty taste for the first time by comparing the nerve responses to NaCl before and after amiloride application in both WT and KO mice. So this study provides convincing data on the effects of Skn1a KO on the amiloride-sensitive salty taste. However, reviewers agreed that the authors need to reconcile the controversy over the salty taste phenotype in the Skn1a KO mice in the Discussion (i.e., why do their findings surpass the others’?)

2. Second, reviewers pointed out that most of the work in this paper was performed with digoxigenin-labeled ISH probes, which is not quantitative. Reviewers agreed that it would be helpful for the authors to perform single cell qRT-PCR from defined TRC subsets (e.g using ENaCa-Cre;Ai9 mice) to determine expression of various marker genes (Skn1a, Calhm3, Trpm5, Pkd2l1 etc.). This would give the field a more comprehensive, (semi) quantitative picture of the different subsets of TRCs. An alternative option would be to perform co-immunostaining of some key components (Skn1a, Calhm3, Trpm5, Pkd2l1 etc.).

3. Finally, reviewers suggest that the authors mention the limitations of their study (e.g. the sensitivity of the assays) in the Discussion.

Reviewers original comments are pasted below:

Reviewer 1:

The main finding of this study is that Calhm3+ TrpM5- is the defining molecular signature of amiloride-sensitive salty TRCs. TrpM5-expression has often been used to characterize sweet, bitter, umami TRCs, and by extension, Type II cells, but this manuscript places salty TRCs in a distinct (TrpM5-) subset of Type II cells.

To characterize TRC subsets, author(s) performed double ISH with known TRC markers. This approach is not comprehensive and is limited by the biochemical eccentricities that affect detection sensitivity. Would such molecular characterization hold up when strong drivers (e.g. TrpM5-Cre) are employed? Other methods to validate the findings, such as RT-PCR, would be useful.

Moreover, while the requirement of Skn1 for production of salty TRCs is a purported finding of the manuscript, numerous studies from Matsumoto et al (2011) to Larson et al (2020) have explored the effects of Skn1 deletion on salt sensation. Although there are some incongruencies (sometimes attributed to methods of anesthesia), recently consensus has been building on the essential role for Skn1 in salty taste. How does identification of Calhm3+ TrpM5- cells explain some of the inconsistencies in the literature?

Efforts to further characterize the Calhm3+ TrpM5- population with ultrastructural or gene expression analysis (as discussed by the authors) would greatly strengthen the paper.

Reviewer 2:

Within taste buds, each of the five basic tastes is sensed by dedicated taste cells. While the molecular features of sweet-, bitter-, umami-, and sour-sensing taste cells have been elucidated, those of salt-sensing taste cells remain unclear. Multiple cell populations are reportedly involved in the perception of salty taste. One constitutes the amiloride-sensitive component found only in the anterior part of the tongue, and the others form the amiloride-insensitive component. Previous studies suggested the taste cells responsible for the amiloride-sensitive salty taste (sodium-taste cells) as ENaCa+/Calhm1+/Calhm3+/Trpm5- cells in fungiform taste buds. In this study, using the in situ hybridization technique, the authors confirmed the existence of such cell population in taste buds in the fungiform papillae and soft palate epithelia, and further characterized the sodium taste cells by elucidating their gene expression profile. The major finding of this study is that the sodium taste cells exhibit a gene expression profile similar but not identical to sweet-, bitter-, umami-sensing type II taste cells, and they rely on Skn-1a for their generation like type II cells. It also provides data supporting the involvement of sour-sensing taste cells in the detection of the amiloride-insensitive salty taste. The findings are substantiated by the loss of amiloride-sensitive gustatory nerve responses to NaCl stimuli in Skn-1a KO mice. The experiments are of high-quality and the data and interpretation are convincing, and the manuscript is well written. Overall, this study provides significant insights into the peripheral taste coding - especially of salty taste - and the development of taste bud cells. However, I have minor comments as described below:

(1) Line 100-101, Line 225, etc.: the background needs to be discussed separately in the fungiform and circumvallate taste buds, as it has been suggested that the variety of taste cells are different between them (e.g. although not directly demonstrated, ENaCa+/Calhm1+/Calhm3+/Trpm5- cells have been suggested in the fungiform but not circumvallate taste buds). So, it needs to be clarified what was demonstrated in which taste buds. This will make the situation clearer to the readers.

(2) Figure 5: The chorda tympani nerve responses are normalized to those to 100 mM NH4Cl, a chloride salt. Do NH4Cl responses involve the AI salty taste cells including bitter cells? If so, knockouts of Calhm3 and Skn-1a might have affected the control responses. It would be helpful if the validity of NH4Cl as the control stimulus is explained.

(3) Typos: line 51, evoking salty taste -> they?; line 109, proposee -> proposed; line253 and many, Trmp5 -> Trpm5. Carefully check the manuscript for typos again.

(4) Fig. S5 -> Fig 6-1
