# Supplementary figures and images for: Sodium–Taste Cells Require Skn-1a for Generation and Share Molecular Features with Sweet, Umami, and Bitter Taste Cells
Source: eNeuro. 2020 Dec 3;7(6):ENEURO.0385-20.2020. doi: 10.1523/ENEURO.0385-20.2020 (PMC7729297; doi:10.1523/ENEURO.0385-20.2020)

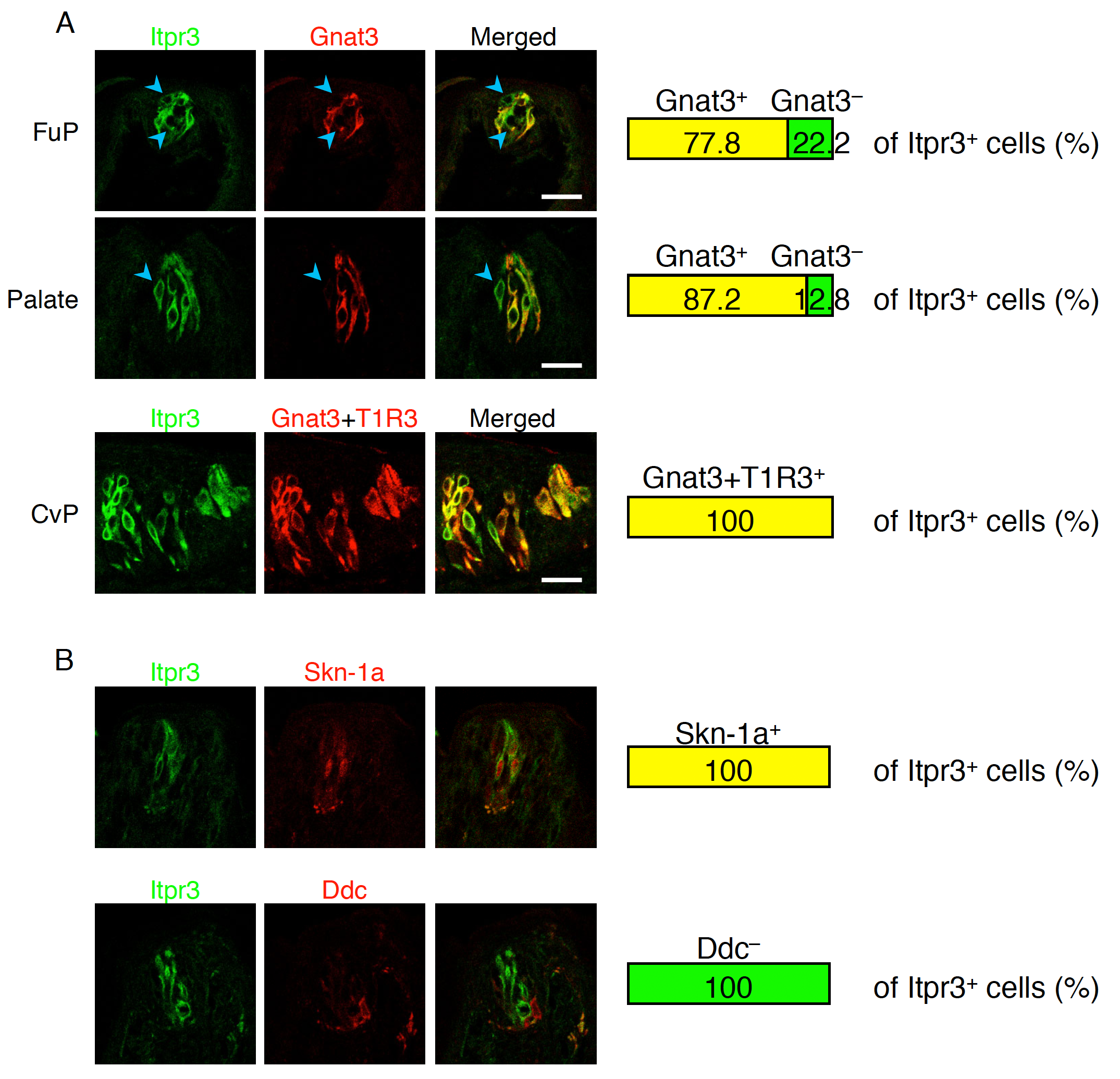

Supplement: Extended Data Figure 1-1 — Immunohistochemical identification of sodium–taste cells. A, Double-fluorescence immunohistochemistry using anti-Itpr3 and anti-Gnat3 antibodies. Itpr3+Gnat3– cells in taste buds of FuP (top) and soft palate (middle) are indicated by blue arrowhead in the merged image (right). In taste buds of CvP (bottom) where cells identified by the expression of Gnat3 and/or T1R3 are equivalent to Trpm5+ cells (Ohmoto et al., 2011), Itpr3+ cells are always positive to Gnat3 and/or T1R3. N = 3. B, Double-fluorescence immunohistochemistry using anti-Itpr3 and anti-Skn-1a (top) or anti-Ddc (bottom) antibodies. Itpr3 signals are present in Skn-1a+ cells and absent in Ddc+ cells. N = 2. Scale bars: 25 μm. Download Figure 1-1, TIF file. [file enu-eN-NWR-0385-20-s01.tif]

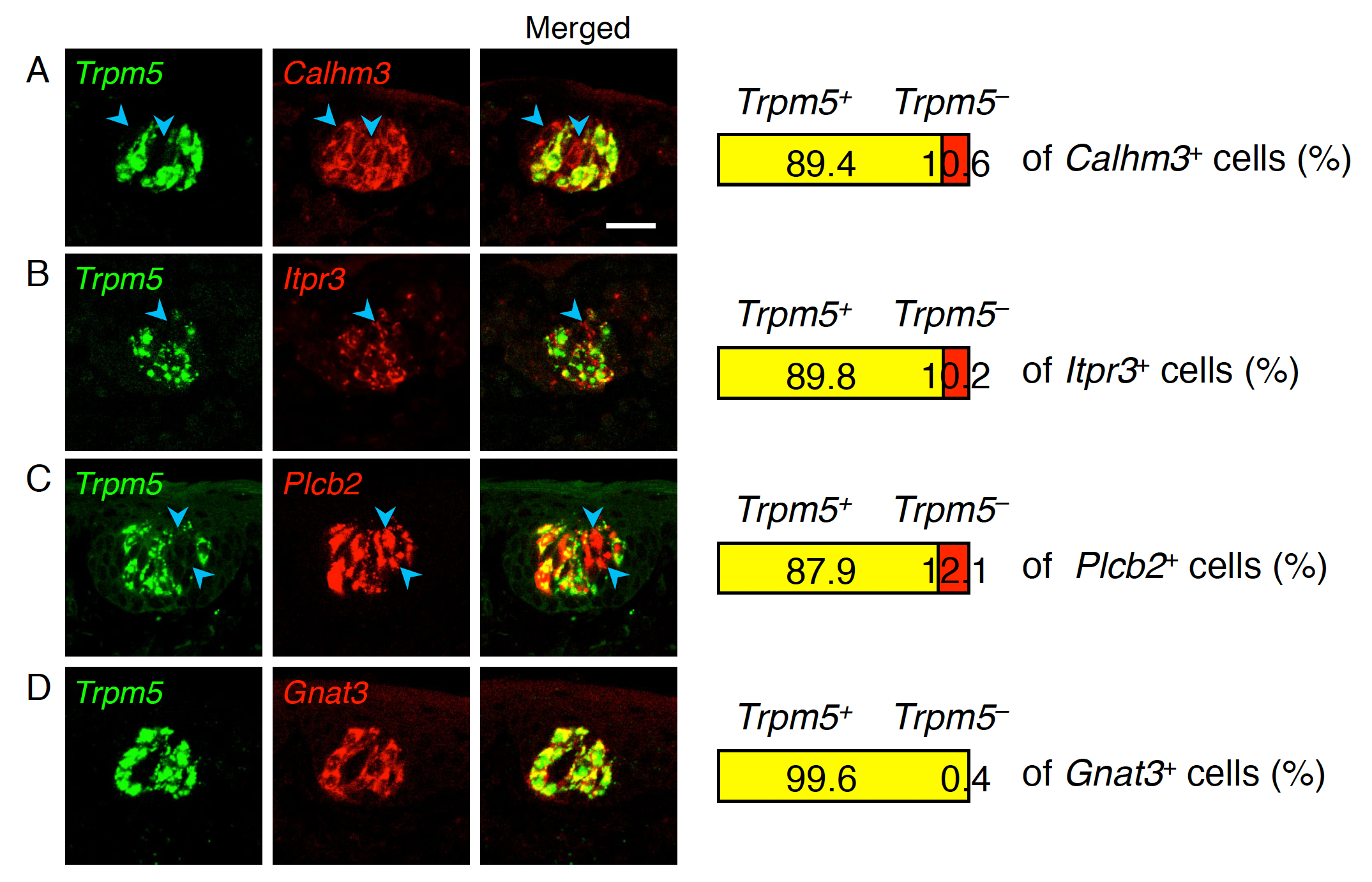

Supplement: Extended Data Figure 1-2 — Expression of taste cell genes in taste buds in soft palate. Double-fluorescence in situ hybridization was performed to study the relationship of expression of Trpm5 with Calhm3 (A), Itpr3 (B), Plcb2 (C), and Gnat3 (D) required for sweet, umami, bitter, or salty taste reception. Numbers of cells showing signals were counted, and the ratios of cells positive and negative for Trpm5 to the total population of cells positive Calhm3 (A), Itpr3 (B), Plcb2 (C), and Gnat3 (D) are shown at the right (n = 3). Blue arrowheads indicate Calhm3, Itpr3, or Plcb2 single-positive cells. Scale bar: 25 μm. Download Figure 1-2, TIF file. [file enu-eN-NWR-0385-20-s02.tif]

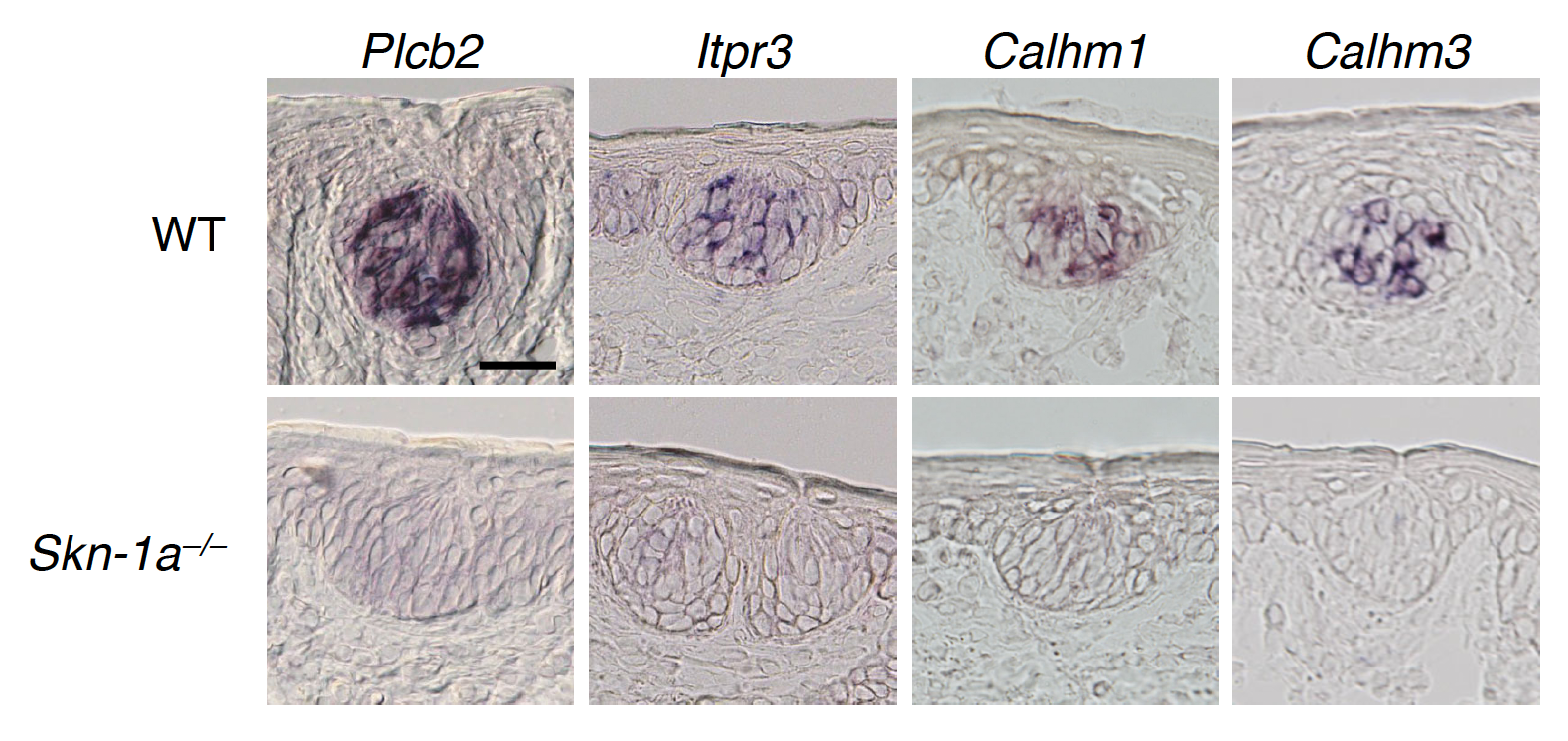

Supplement: Extended Data Figure 2-1 — Requirement of Skn-1a for the expression of Plcb2, Itpr3, Calhm1, and Calhm3 in taste buds in soft palate. In situ hybridization analyses revealed that the expression of Plcb2, Itpr3, Calhm1, and Calhm3 observed in WT mice (top) were not detected in taste buds in Skn-1a–/– mice (bottom). Scale bar: 25 μm. Download Figure 2-1, TIF file. [file enu-eN-NWR-0385-20-s03.tif]

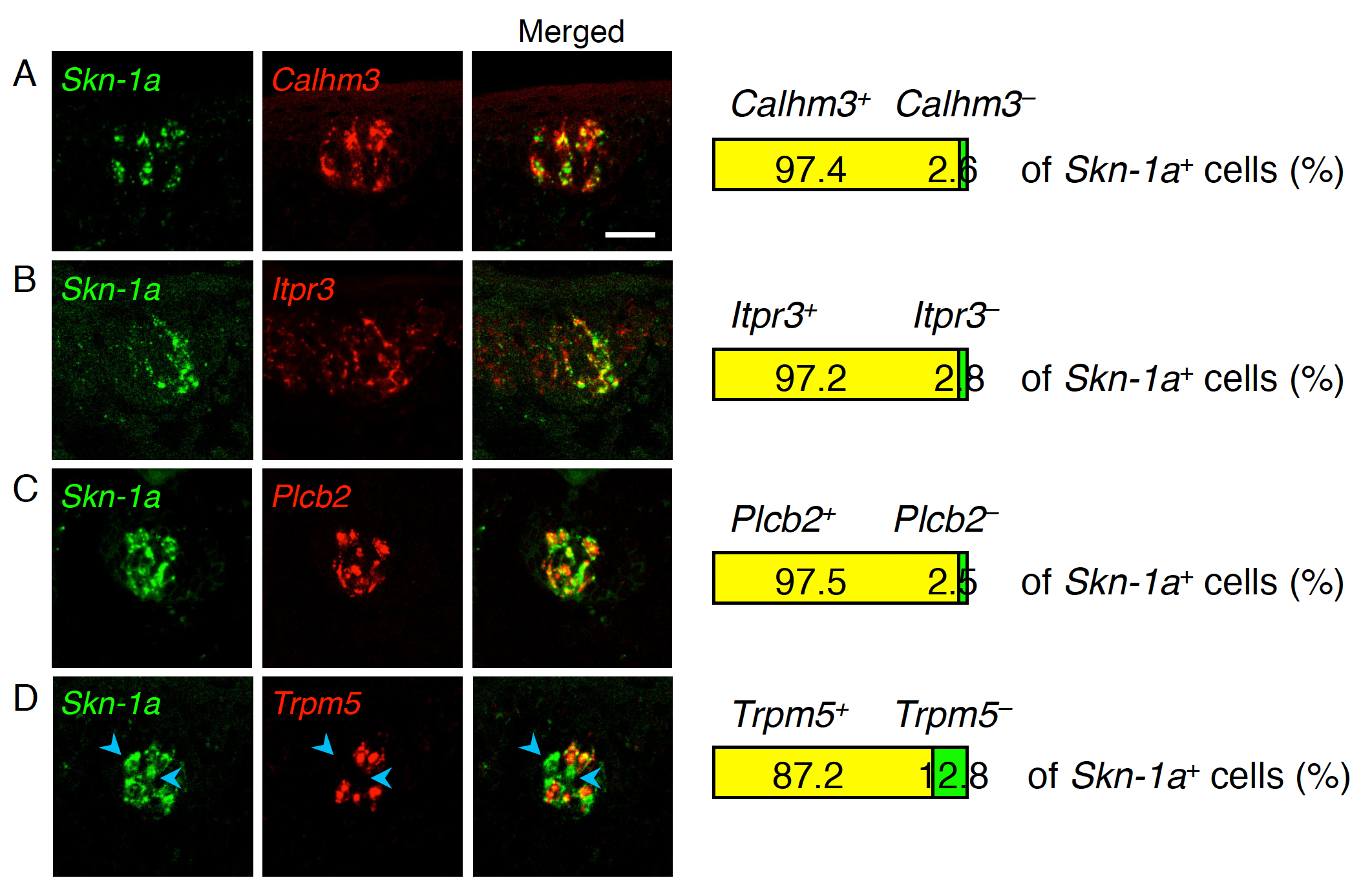

Supplement: Extended Data Figure 3-1 — Co-expression of Skn-1a with taste cell genes in taste buds in soft palate. Double-fluorescence in situ hybridization was performed to study the relationship of expression of Skn-1a with Calhm3 (A), Itpr3 (B), Plcb2 (C), and Trpm5 (D). Numbers of cells showing signals were counted, and the ratios of cells positive and negative for Trpm5 to the total population of cells positive Calhm3 (A), Itpr3 (B), Plcb2 (C), and Trpm5 (D) are shown at the right (n = 3). Blue arrowheads indicate Skn-1a single-positive cells. Scale bar: 25 μm. Download Figure 3-1, TIF file. [file enu-eN-NWR-0385-20-s04.tif]

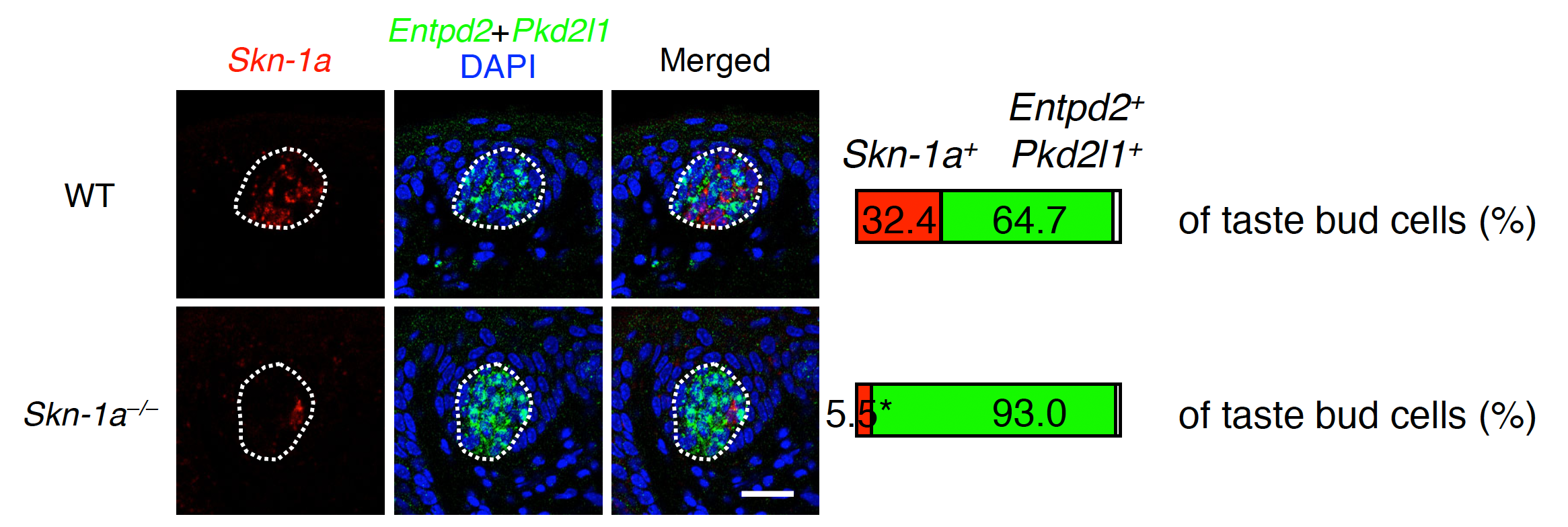

Supplement: Extended Data Figure 4-1 — Disappearance of Skn-1a-dependent taste bud cells by Skn-1a deficiency. Populations of Skn-1a+ and Skn-1a– cells (i.e., positive to a mixed probe to Entpd2 and Pkd2l1) in taste buds in soft palate were quantified by double-fluorescence in situ hybridization analyses. Taste bud profiles are outlined by broken white lines. Asterisk indicates the ratio expressing mutant Skn-1a mRNA. Decrease of Skn-1a+ cell population was statistically evaluated by Welch’s t test: p = 0.0001. Scale bar: 25 μm. Download Figure 4-1, TIF file. [file enu-eN-NWR-0385-20-s05.tif]

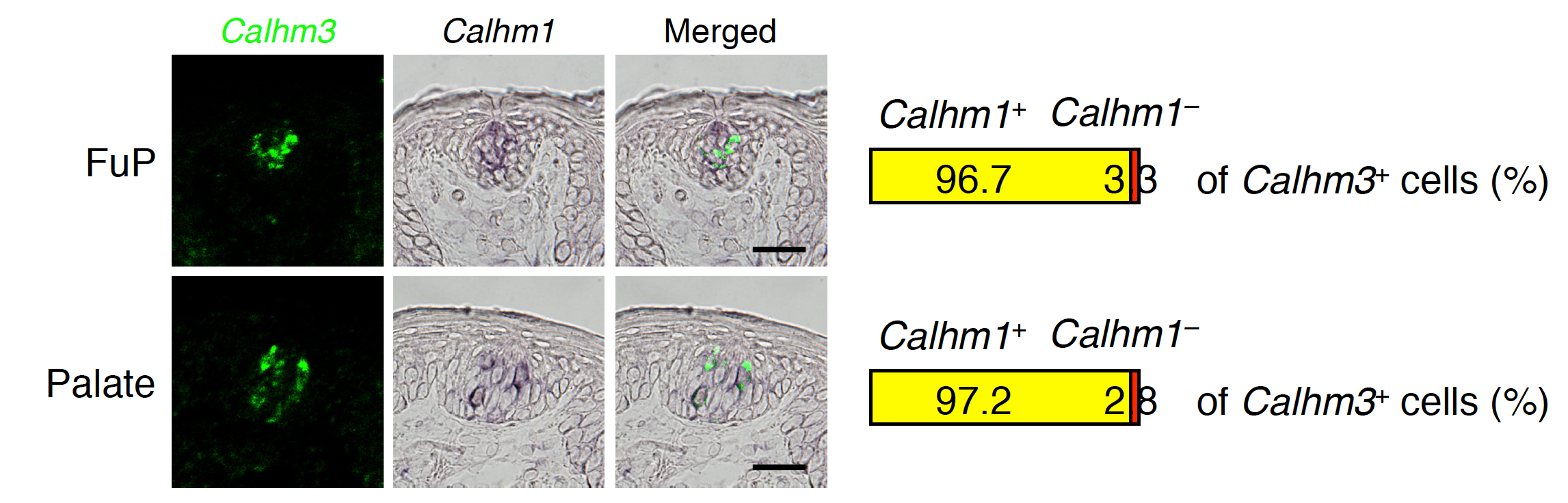

Supplement: Extended Data Figure 6-1 — Co-expression of Calhm1 and Calhm3 in taste buds. Double-fluorescence in situ hybridization was performed to study the relationship of expression of Calhm1 with Calhm3. Numbers of cells showing signals were counted, and the ratios of cells positive and negative for Calhm1 to the total population of cells positive Calhm3 are shown at the right (n = 3). Scale bars: 25 μm. Download Figure 6-1, TIF file. [file enu-eN-NWR-0385-20-s06.tif]

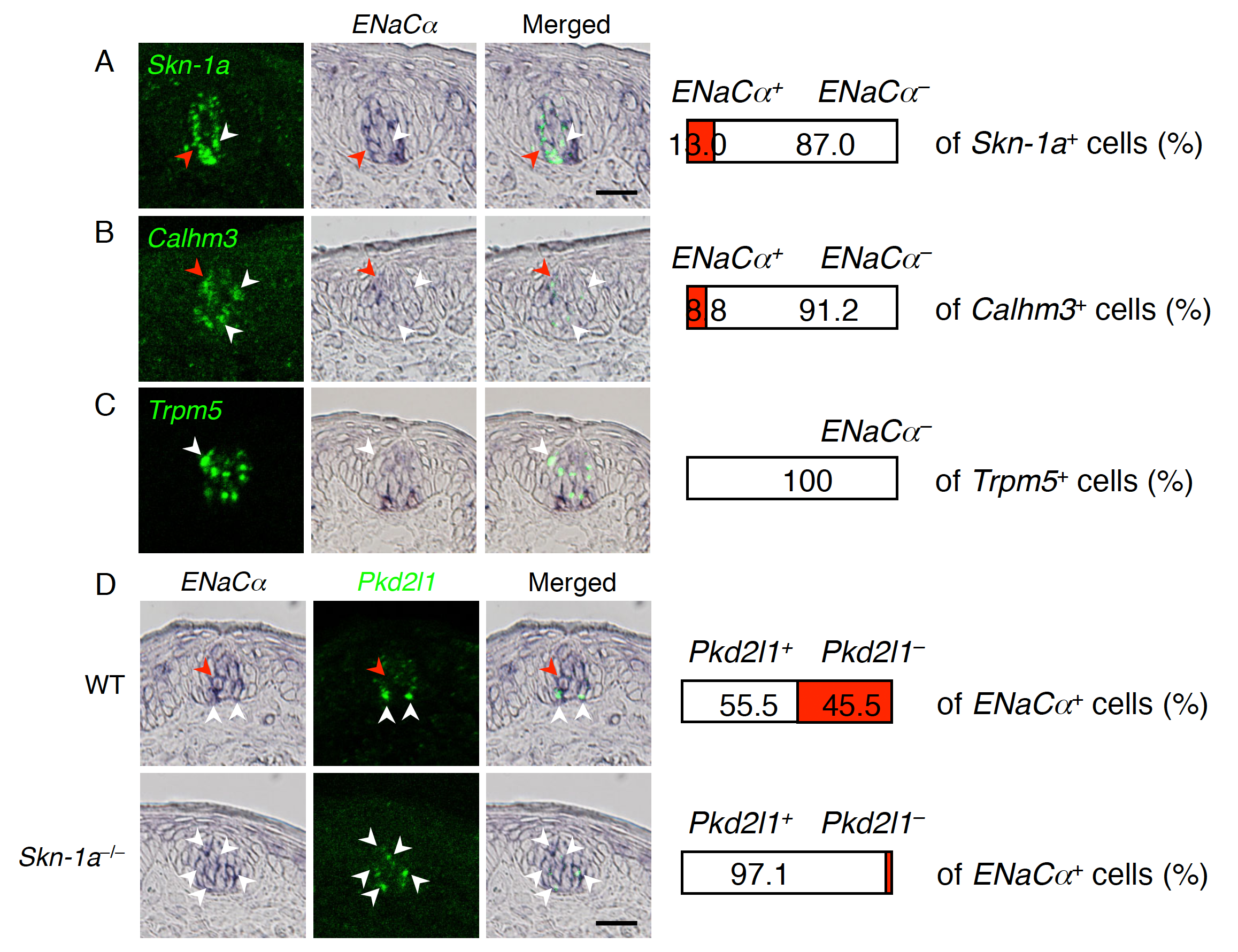

Supplement: Extended Data Figure 6-2 — ENaCα expression in Calhm3+Trpm5– sodium–taste cells in soft palate. Double-labeling in situ hybridization was performed to study expression of ENaCα in Calhm3+Trpm5– sodium–taste cells. A–C, ENaCα expression and that of Skn-1a (A), Calhm3 (B), and Trpm5 (C). Numbers of cells showing signals were counted, and the ratios of cells positive and negative for ENaCα (middle images) to the total population of cells positive for the gene (left images) are shown at the right (n = 3). White arrowheads indicate Skn-1a, Calhm3, or Trpm5 single-positive cells, and red arrowheads indicate cells co-expressing ENaCα with Skn-1a, Calhm3, or Trpm5. D, Robust decrease of ENaCα-expression in non-sour taste cells by Skn-1a deficiency in taste buds. Populations of Pkd2l1+ and Pkd2l1– cells in ENaCα-expressing cells were quantified by double-labeling in situ hybridization analyses. White and red arrowheads indicate representative Pkd2l1+ENaCα+ and Pkd2l1–ENaCα+ cells, respectively. Decrease of the Pkd2l1–ENaCα+ cell population was evaluated by Welch’s t test: p = 0.0009. Scale bars: 25 μm. Download Figure 6-2, TIF file. [file enu-eN-NWR-0385-20-s07.tif]
